# Current *versus* future reproductive investment adaptive responses in adult *Anopheles coluzzii* malaria mosquitoes: hydric-stressed males give it all

**DOI:** 10.1186/s13071-019-3608-3

**Published:** 2019-07-30

**Authors:** Nkiru E. Ekechukwu, Frédéric Tripet

**Affiliations:** 1 Centre for Applied Entomology and Parasitology, School of Life Sciences, Keele University, Newcastle-under-Lyme, Staffordshire ST5 5BG UK; 2 Department of Zoology and Environmental Biology, Faculty of Biological Sciences, University of Nigeria Nsukka, Nsukka, Nigeria

**Keywords:** Trade off, Reproductive value, Hydric stress, Mating, Sperm quantification, qPCR, Sperm activity, *Anopheles gambiae*

## Abstract

**Background:**

Life history theory predicts that an individualʼs current reproductive investment should depend on its future reproductive value. A variety of intrinsic biotic and extrinsic factors influence reproductive value, including age, health status and current environmental conditions. Phenotypic plastic reproductive decisions are particularly crucial in species with limited mating and breeding opportunities. In the malaria mosquito *Anopheles coluzzii*, the combination of male-male competition and female monandry results in male reproductive success being dependent on limited mating opportunities and sperm reserves. Short life spans combined with 3–4 day gonotrophic cycles imply that females can produce only a limited number of egg-batches in their lifetime and rely on a single maleʼs insemination to do so. Here we experimentally tested the effect of hydric stress on male sperm transfer and female sperm maintenance in this important vector species.

**Methods:**

Virgin males and females were stressed prior to mating to simulate environmental uncertainty, hence the prospect of a decreased lifespan. They were then paired overnight with non-stressed mates in standardized mating assays. Sperm transfer, uptake and maintenance were quantified using qPCR, and sperm activity determined *via* video recording.

**Results:**

When exposed to hydric stress, males responded by increasing their current reproductive investment and transferred significantly larger amounts of sperm to females. There was no significant increase in the mean number of females inseminated overnight by stressed males. In contrast, females did not significantly change their sperm uptake following stress nor did they alter their sperm maintenance strategy after 7-day post-mating hydric stress as measured through sperm activity level and sperm cells quantification.

**Conclusions:**

As predicted by life-history theory, pre-mating hydric stress was associated with an increase in male current reproductive effort in the form of increased sperm transfer. In contrast, pre and post-mating hydric stress had no impact on sperm uptake and maintenance by females, which is compatible with the prediction that females maximize their reproductive value by withstanding stress periods until a blood meal opportunity and maintain sperm quality towards future egg production.

## Background

Life history trade-offs, such as that between current and future reproduction, are crucial for understanding phenotypic plastic responses of living organisms to their environments. Theoretical models show that both current and future reproductive success depend on current investment in reproduction [1, 2]. Empirical studies have demonstrated this important trade-off by measuring the negative impact of an increase in current reproductive effort on survival and future reproduction. Depending on their life-cycle, parasites can affect the costs of current or future reproduction differentially [3–5]. Thus, experimental parasitic infections have been instrumental in demonstrating how animals can adjust their investment in current reproduction, their survival and future reproduction to maximize their lifetime reproductive success [5–7]. As an example, adult blue tits, *Parus caeruleus,* with nests experimentally infested by hen fleas, *Ceratophyllus gallinae*, compensated for the fleasʼ negative effects on nestling growth by increasing nestling feeding rates [6]. This increase in current reproductive effort resulted in decreased adult survival and smaller egg-batches the following year [7]. In contrast, *Biomphalaria glabrata* snails infected with *Schistosoma mansoni*, a trematode known to shorten its hostʼs lifespan, increased egg production upon experimental infection [5].

In addition to an organismʼs health status, a variety of other intrinsic and extrinsic factors can influence the balance between current and future reproductive value, including sex, age, sexual competition and various environmental stressors. The trade-off can sometimes be affected in a sex-dependent manner, resulting in distinct phenotypic responses in males and females; this has been demonstrated in a variety of vertebrates and invertebrates [5, 8]. In mating systems in which females are the limiting sex, females are predicted to invest less than males in costly reproductive behaviour and should therefore maintain behavioural and physiological responses that promote survival. In contrast, males should prioritize sexual activities that increase their chances of current reproductive success but may decrease their future reproductive value [9, 10]. These include mate-finding activities, investment in sperm production, and insemination, all of which are costly for survival and future reproductive success [11–14]. In male *Drosophila nigrospiracula* fruit flies, infection with the parasitic mite *Macrocheles subbadius* ultimately results in decreased lifespan [15]. When experimentally infected, males responded by increasing their courtship behaviour and mating rates [15]. In the wood frogs *Lithobates sylvaticus,* males and females differed in their response to handling stress, which was used as a proxy for predation threat [16]. Following handling, males increased their investment in risky mate-finding behaviour which is conspicuous to predators, whilst females increased those promoting survival and future reproduction [16].

In the Culicidae, very little is known of the trade-off between current and future reproduction. Millions of mosquitoes are being produced weekly for disease control programmes, relying on releases of sterility-inducing males [17] or *Wolbachia*-carrying strains [18]. A lot of research has focused on the impact that rearing conditions such as diet, density and environmental factors such as temperature and humidity have on male survival and mating competitiveness. However, the possible interactive effect that stressors such as, for example, hydric stress have on the trade-off between current *versus* future reproduction is unknown. Many mosquito species, including *Anopheles coluzzii*, one of the most important vectors of malaria in West-Africa, live in semi-arid regions. These habitats are characterized by strong seasonal variations in temperature, relative humidity and rainfall, which determine the occurrence and seasonal abundance of *An. coluzzii* [19–21]. In addition to seasonal droughts, mosquito populations in the Sahelian belt are subjected to strong daily fluctuations in temperature and relative humidity. These persistent environmental fluctuations make adaptations for coping with hydric stress and maintaining an adequate body water balance some of the most important aspects of the physiology of *An. coluzzii* [21, 22]. The average lifespan of this species is only a few days and most females will lay only one or two egg-batches in their lifetime. Females are also largely monandrous and visit swarms initiated by males at dusk once in their lifetime to mate [23]. Males can swarm for several consecutive nights but are not capable of inseminating more than three females per night because of physiological constraints on sperm and mating plug production [24]. Depending on the season, swarms can involve tens or hundreds of individuals and females contribute a small proportion of that number [25]. In the swarm mating system, females are clearly the limiting sex and sexual selection on males is particularly intense with approximately 7% of males successfully pairing on any given night [25]. Field and laboratory studies have also shown that females mated more frequently with males of intermediate-to-large size and 4–8 day-old males [26, 27]. Laboratory and field experiments have also established that mating carries large energetic costs which translate in decreased future survival in males which swarm for extended periods of time [26, 28]; but this is not the case in females which become refractory to further mating attempts following successful insemination [23]. In the anophelines, males transfer a mating plug along with the sperm and this sticky mass fills the female atrium and acts as a physical barrier preventing females from re-mating [23, 29]. Importantly, the plug also acts as vehicle for different important compounds responsible for a signalling cascade leading to sperm uptake, refractoriness to further mating, and the initiation of ovogenesis [29, 30].

Here we tested the hypothesis that hydric stress may differentially affect the trade-off between current and future reproduction in male and female *An. coluzzii*. We focused on male sperm production and transfer and female sperm uptake and maintenance. These are reproductive traits crucial to the current and future reproductive effort and success of both sexes and have not yet been studied. Males subjected to a life-threatening bout of hydric stress were predicted to transfer larger ejaculates to females, an increase in current reproductive effort that would maximize their lifetime reproductive value given their uncertain survival prospects. Conversely, females undergoing hydric stress could potentially decrease sperm uptake to promote body maintenance and survival, or alternatively, they could strive to withstand harsh environmental conditions to preserve sperm quality towards future egg-batches. Understanding such trade-offs might highlight important novel aspects of male and female reproductive biology with significant implications for vector control programmes in which male mating competitiveness and sperm transfer play an important role.

## Methods

### Mosquito rearing

All experiments were conducted in 2011 and 2012 at the Centre for Applied Entomology and Parasitology, Keele University, UK. The mosquitoes used were of the ‘Mopti 2003ʼ strain, an 8 year-old wild-type strain of *An. coluzzii* colonized from the village of N’Gabakoro Droit in Mali, West Africa in 2003, obtained from the Reference Reagent Resource Centre (MR4) and maintained in dedicated insectaries as described in previous studies [21, 31]. The Mopti strain is of the so-called Mopti chromosomal form, characterized by the *bc* and *u* inversion on chromosome 2R and fixed for inversion *a* on 2L [19, 32]. Mosquitoes were kept at 27 ± 2 °C, 70 ± 5% relative humidity, with a 12 h light/dark photocycle. To ensure similar phenotypic quality across all experiments, larvae were grown at a density of 200 larvae/l and fed on ground Tetramin fish food (Tetra, Melle, Germany) [21]. Upon pupation, pupae were transferred to a standard rearing cage made of a 5 l white polypropylene bucket (~ 20.5 cm height × 20 cm diameter) with a sleeved side opening for introducing and removing mosquitoes and accessories, and the top covered with mosquito netting. Adults were maintained at densities of 600–800 adults per enclosure and provided with cotton wool pads soaked in water on top of cages and 5% glucose solution *via* dispensers inside the cages. Females were blood fed using horse blood provided *via* a Hemotek membrane feeding system (Discovery Workshops, Accrington, UK).

### Experimental design

#### Pre-mating hydric stress and sperm transfer by males

The effect of hydric stress prior to mating on male reproductive investment was tested by quantifying sperm transfer and insemination rate. Before mating overnight, 50 randomly picked 2–4-day-old virgin *An. coluzzii* males were transferred to a mating cage and kept under standard insectary conditions (control or no stress). Two other mating cages (50 virgin males per cage) were set up and males from one cage were stressed for 4 h (8:00–12:00 h) and males from the other cage for 8 h (8:00–16:00 h). Stress was induced by substituting the 5% glucose solution in the cages with 2 cubes of sugar and by replacing the wet cotton wool pads on top of the cages with dry ones. At the end of the stress period, the stressed cages received glucose solution until 18:00 h, at which stage the cages of males from the 3 treatment groups (controls, 4-h and 8-h stress) were combined with a hundred 2–4 day-old non-stressed virgin *An. coluzzii* females for overnight mating (1:2 male/female ratio). The design ensured that the mean age of males and females at mating was identical across all 3 experimental groups and that mating overnight was staged at exactly the same time for all groups. The following morning (6:00 h), all females were collected stored in 75% ethanol and later dissected to determine their insemination status and for subsequent sperm quantification. The experiment was duplicated using independent successive mosquito cohorts.

#### Pre-mating hydric stress and sperm uptake by females

A reciprocal of the hydric stress experiment was conducted on females to assess the effect of hydric stress on sperm uptake prior to mating. The setup was identical to the male stress experiment. Randomly-picked virgin *An. coluzzii* females were transferred to 3 mating cages (100 per cage). The first cage was kept under normal conditions and the other two cages were stressed as described above. After stressing, the cages of females from the 3 hydric stress treatment groups (0-h, 4-h and 8-h stress) were combined with 50 non-stressed virgin *An. coluzzii* males for overnight mating. The following morning, all females were collected, stored in 75% ethanol and dissected to determine their insemination status and for subsequent sperm quantification. The experiment was conducted in duplicates using successive mosquito cohorts.

#### Post mating hydric stress and sperm maintenance by females

The effect of hydric stress on sperm activity and sperm storage in mated *An. coluzzii* females was explored by subsequently providing limited access to water to the females seven days post-mating. Here, sperm activity in the spermathecae was used as a proxy for the proportion of live sperm stored by females as higher sperm activity is an important correlate of fertility in vertebrate and insect taxa [33, 34]. First, 2–4 days-old virgin females were combined with same age virgin males in 3 mating cages for overnight mating. To ensure that all the females were mated before the next morning, excess males were provided in a ratio of 1:2 (50 females and 100 males per cage). The cages were supplied with 5% glucose solution for food and wet cotton pad for water. The following morning, males were removed from the cages. The females in the first cage were not stressed after the mating period and had access to water and 5% sugar solution as before, whereas the females in the other two cages were stressed. The stress was performed by replacing 5% sugar solution with 2 sugar cubes and swapping the moist cotton pads with dry ones on top of each cage for 4 h (14:00–18:00 h) and 8 h (10:00–18:00 h) respectively. After the stress period, water and 5% sugar solution were provided as before. These exchanges were made daily for 7 days, following which, a random subset of females was collected for quantification of sperm activity, and the others were collected stored in 75% ethanol then later dissected to determine their insemination status and sperm quantification. The experiment was duplicated using successive mosquito cohorts.

### Biological analysis

#### Male and female body size measurement

The wing length of females was measured as an accurate correlate of their body size [35]. Wings were measured from the alular notch to the distal wing margin, excluding the fringe scales, to the nearest 0.01 mm using a binocular microscope with an eyepiece graticule. The number of females found alive and dead at the end of stress experiments was recorded.

#### Determination of insemination status

For sperm quantification, the female spermathecae were dissected under a binocular dissecting microscope (Leica Microsystems GmbH, Solms, Germany) as described in previous studies [36, 37]. Presence of the sperm bundle in the spermatheca indicates a mated female while absence of the sperm bundle implies no mating [36].

#### Sperm quantification

In females with sperm present in their spermatheca, the sperm bundle was gently picked up with a dissecting pin and transferred to 500 µl of lysis mix combined with 10 µl of proteinase K and incubated overnight on the heat block at 55 °C. The next morning, DNA was extracted from it using a ChargeSwitch gDNA micro tissue kit (Invitrogen, Carlsbad, CA, USA) following the manufacturer’s instructions.

Sperm quantification was performed as described previously [24]. The number of sperm cells contained in the gDNA extracts from the spermathecae was estimated using a Taqman assay (Applied Biosystems, Foster City, CA, USA) targeting a known Y-chromosome-specific sequence [38]. The following primer and probe were used: forward (5′-TTA CCA CGC TGG CAA ATG C-3′); reverse (5′-CGT GCA ACA GCT CGT GAT G-3′); probe (5′-ACG CCG CAT CCA TCT-3′). Quantitative real-time PCRs were run using TaqMan Universal Master Mix (Applied Biosystems) on a Step-One-Plus Real Time PCR System (Applied Biosystems Foster City, CA, USA). PCR steps were: 50 °C for 2 min, 95 °C for 10 min, 95 °C for 0.15 s, and 60 °C for 1 min, for 30 cycles. Cycle threshold values (CT) were translated into number of Y-chromosome copies using the standard curve method and a pool of male mosquito DNA as standard. The concentration of the pool of male DNA was first adjusted to 50,000 haploid genome copies/ml using a theoretical approximate molecular weight of 0.3 pg per genome, i.e. 1.96e−21 g/bp and 278,253,050 bp [39]. Two independent five-step serial dilutions curves were made per PCR plate. The curve with the best fit was used to translate CT-values into estimated numbers of Y-chromosomes/ml, which were multiplied by two to account for female sperm (assuming a 1:1 male to female sperm ratio) and multiplied by the total volume of the DNA extraction from inseminated spermathecae (75 ml).

#### Sperm activity

At the end of the 7-day post-mating stress experiment, females were collected for sperm activity determination as described in [24]. A random subset of 6 females obtained from each treatment cage and each replicate was briefly knocked down on ice and swiftly dissected in PBS buffer under a binocular microscope. Using a pair of microneedles, the last abdominal segment of the mosquito was opened and the intact spermathecae separated from the abdomen. Recordings were made by promptly transferring the microscope slides under an inverted phase-contrast microscope equipped with a digital video camera (Motic China Group Co Ltd, Xiamen, China) with light shining through the spermathecal wall and 200× magnification. The video recordings were visualized at a later stage and the sperm movement scored every 10 s during the 1 min of recording and ranked as 0 (no activity), 1 (very low), 2 (low), 3 (medium), and 4 (high). Scoring of sperm activity was done ‘blindly’ with regard to the experimental treatment. Scoring was made by trained observers, a method that led to high repeatability in preliminary experiments.

### Statistical analysis

All analyses were carried out using JMP 10.0 software (SAS Institute, Inc. USA). The datasets were inspected for normality in distribution and heterogeneity of variance. Frequency data were compared *via* a Pearsonʼs chi-square test. Parametric continuous data were analysed using ANOVA and are reported and displayed as means with 95% confidence intervals throughout. *Post-hoc* pairwise comparisons were tested using Tukeyʼs test. Sperm data were log-transformed for parametric analyses. Sperm vigor scores and other continuous non-parametric data were compared using Mann–Whitney and Kruskall-Wallis tests and reported as median and quartiles and displayed as boxplots. Replicate effects were tested in all analyses but are only reported when significant.

## Results

### Effect of pre-mating hydric stress on male current reproductive effort

**Fig. 1:**
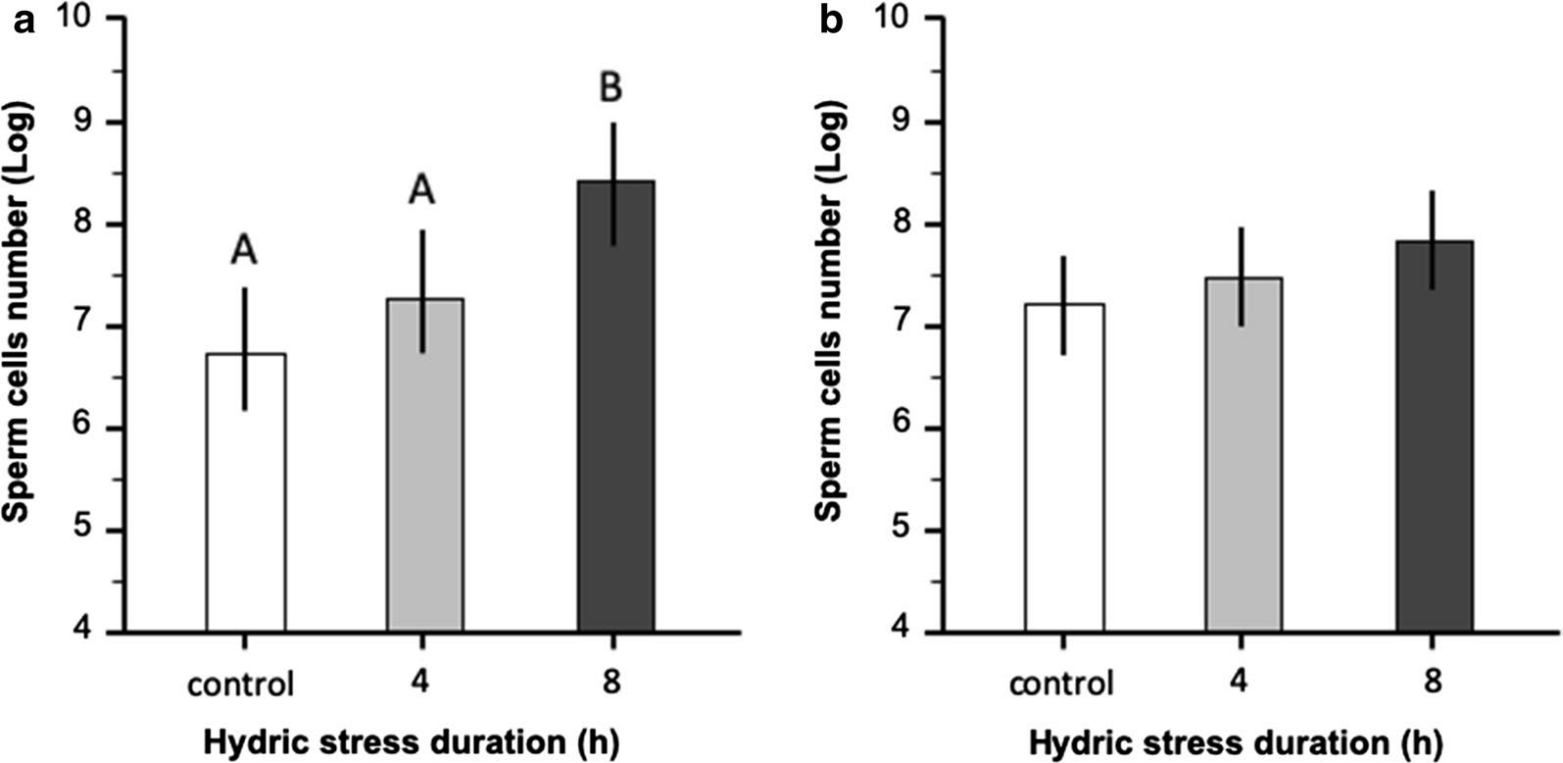
Number of sperm cells (log-transformed) quantified from female spermathecae in the pre-mating hydric-stressed male experiment (**a**), and the pre-mating stressed-female reciprocal experiment (**b**). Males or females were stressed prior to overnight mating by preventing access to water for stress durations 4 h and 8 h or no stress (control). Means and 95% confidence intervals (error bars) are presented and significant differences (*post-hoc* Tukeyʼs tests) are indicated by different letters

**Fig. 2:**
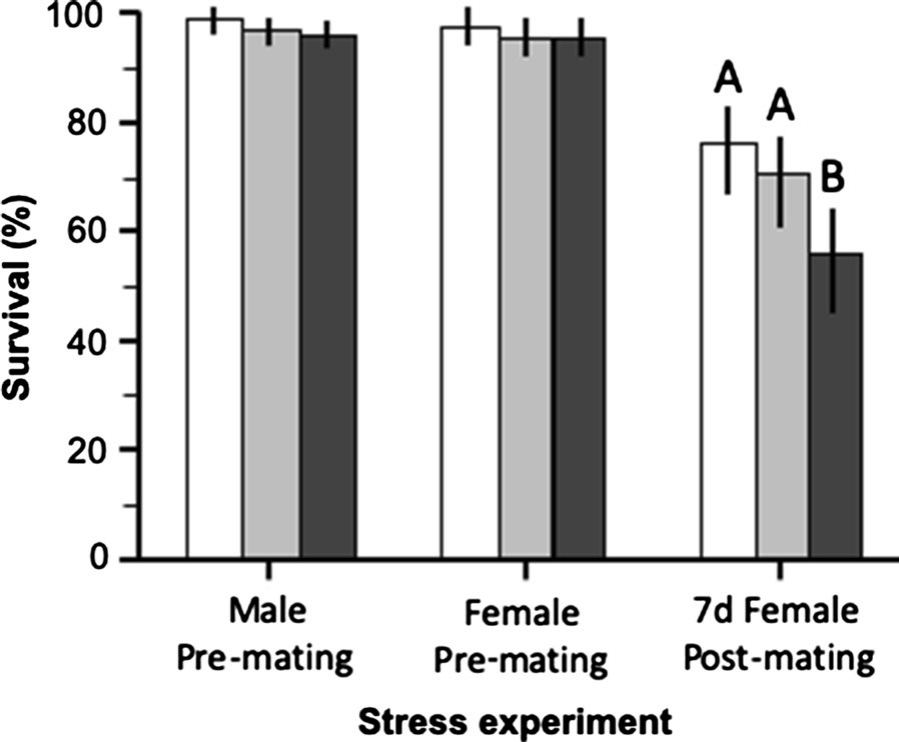
The percent of female surviving across hydric stress treatment groups in the three hydric stress experiments Survival (percentages) are presented along with 95% confidence intervals (error bars) and significant differences (Chi-square Likelihood Odds-Ratios) are indicated by different letters

Across all hydric stress treatment groups, an average of 52.6% of virgin females were inseminated by males overnight and no significant difference was observed in insemination frequency among treatment groups (*χ*^2^ = 0.04, *df* = 2, *P* = 0.978, *n* = 587). In contrast, the level of hydric stress strongly affected the amount of sperm cells transferred to females by males (ANOVA: *F*_(2,117)_ = 58.2, *P* < 0.001) (Table 1). Eight-hours-stressed males transferred on average twice as many sperm cells as 4-h hydric-stressed males and 3.7 times as much sperm as non-stressed males (Tukeyʼs tests: *n* = 79, *P* = 0.02 and *P* < 0.001, respectively) (Fig. 1a). There was no significant difference in the body size (ANOVA: *F*_(2,117)_ = 0.67, *P* = 0.504) nor in survival rates (*χ*^2^ = 4.58, *df* = 2, *P* = 0.101, *n* = 587) (Table 2 and Fig. 2) of mated females among treatments. Overall, there was no relationship between the amount of sperm (log-transformed data) estimated from the female spermathecae and female body size (regression: *t* = 1.90, *P* = 0.060, *n* = 118).

**Table 1:** The mean estimated sperm cells number measured in the three stress experimental groups (male pre-mating, female pre-mating and 7 day female post-mating)

Treatment group	Control	4 h	8 h
Male pre-mating	2856.46 (1516.09–4196.82) (*n* = 40)	5303.06 (3009.58–7596.54) (*n* = 39)	10597.94 (7307.84–14000.32) (*n* = 39)
Female pre-mating	3086.98 (1700.93–4473.04) (*n* = 39)	4845.02 (2575.39–7114.65) (*n* = 40)	4658.06 (3211.87–6100.25) (*n* = 40)
7 day female post-mating	780.41 (462.85–1097.97) (*n* = 40)	1007.03 (559.64–1454.42) (*n* = 39)	1439.64 (964.19–1915.11) (*n* = 39)

*Note*: Values in parentheses are 95% confidence intervals

*Abbreviation*: n, total sample size

**Table 2:** The percentage survival of females in the three stress experiments (male-pre-mating, female pre-mating and 7 day female post-mating)

Treatment group	Control	4 h	8 h
Male pre-mating	99.50 (99.91–97.21) (*n* = 199)	97.44 (98.90–94.14) (*n* = 195)	96.37 (98.23–92.70) (*n* = 193)
Female pre-mating	97.96 (99.21–94.87) (*n* = 196)	95.89 (97.99–92.08) (*n* = 194)	96.37 (98.23–92.70) (*n* = 193)
7 day female post-mating	76.86 (83.35–68.59) (*n* = 121)	70.59 (78.55–61.13) (*n* = 102)	55.00 (64.39–45.25) (*n* = 100)

*Note*: Values in parentheses are 95% confidence intervals and total sample sizes are in italics

*Abbreviation*: n, total sample size

### Effect of pre-mating hydric stress on female current reproductive effort

Across all treatment groups, an average of 40.8% of virgin females were inseminated by the 50 males overnight which was significantly lower than the insemination rate in the stressed-males experiment (*χ*^2^ = 19.9, *df* = 2, *P* < 0.001, *n* = 1168). Insemination rates were 34%, 40.5 % and 44 % in controls, 4h, and 8h, however, this increase was not statistically significant (*χ*^2^ = 5.0, *df* = 2, *P* = 0.081, *n* = 581). There was no effect of pre-mating hydric stress level on sperm cells uptake in the spermatheca by females (ANOVA: treatment: *F*_(2,116)_ = 3.7, *P*= 0.184, *n* = 118) (Table 1 and Fig. 1b). Females from the three experimental groups did not significantly differ in body size (ANOVA: *F*_(2,117)_ = 0.67, *P* = 0.504) nor in survival rate (*χ*^2^ = 1.47, *df* = 2, *P*= 0.481, *n* = 583) (Table 2 and Fig. 2). There was no relationship between the amount of sperm cells (log-transformed data) in the female spermathecae and female body size (regression: *t* = − 0.88, *P* = 0.381, *n* = 119).

### Effect of 7-day post-mating hydric stress on sperm activity in the spermatheca

**Fig. 3:**
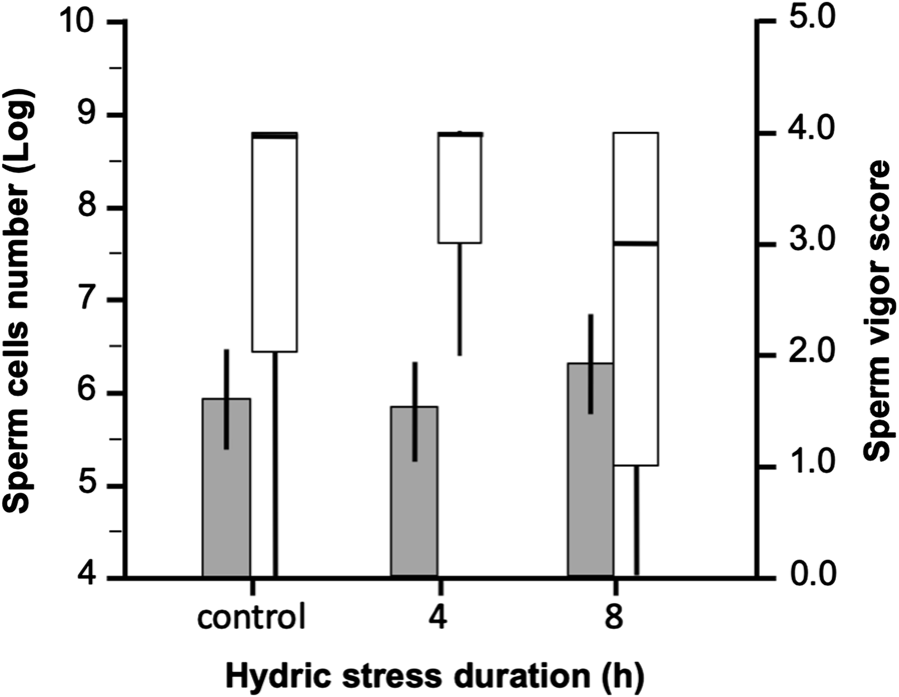
The number of sperm cells (log-transformed) and sperm vigour quantified from female spermathecae in the 7-day post-mating stress experiment. Mean sperm cell numbers (grey bars) are presented with 95% confidence intervals (error bars) and vigour scores (white bars) are shown as boxplots indicating medians and 1st and 3rd quartiles and whiskers (quartiles ± 1.5*interquartile range)

Female insemination rate was 100% across all hydric stress groups. On average, there were 4 times fewer sperm cells in the spermathecae of females 7 days post-mating compared to mated females in the pre-mating stress experiment (Mann–Whitney test: *Z* = 6.67, *P* < 0.001, *n* = 238), and as much as 6 times fewer sperm cells compared to mated females from the male pre-mating stress experiment (Mann–Whitney test: *Z* = 5.48, *P* < 0.001, *n* = 237) (Table 1). There was no significant effect of 7-day post-mating stress level on the estimated number of sperm cells (log transformed data) maintained by females (ANOVA: treatment: *F*_(2,116)_ = 2.53, *P* = 0.415) (Fig. 3). The median sperm activity score was 3 (medium activity) in 8 h-stressed females compared to 4 (high activity) in the control and 4 h-stressed female groups (Kruskall-Wallis test: *H* = 4.82, *P* = 0.0897, *n* = 120) (Fig. 3). There was a significant decrease in female survival rates in relation to hydric stress level (*χ*^2^ = 12.5; *df* = 2, *P* = 0.002, *n* = 323) (Table 2 and Fig. 2). Females did not significantly differ in body size among treatments (ANOVA: *F*_(2,119)_ = 0.23, *P* = 0.796) and there was no relationship between female body size and the number of sperm cells (log-transformed data) estimated from their spermathecae (regression: *t* = 0.16, *P* = 0.877, *n* = 119).

## Discussion

In animals, the decline in longevity typically associated with increased current reproductive effort has often been used to highlight the cost of reproduction [11–13]; and this life-history trade-off is known to be affected by environmental stress [14, 40]. Sperm production and allocation by males, as well as sperm uptake and maintenance by females, are important determinants of their current and future reproductive success. Here, we demonstrated that the physiological stress associated with water scarcity in the malaria mosquito *An. coluzzii* [21, 22] affects how males and females ‘manageʼ sperm. The results of the pre-mating hydric stress studies suggest that males respond adaptively to stress by investing more in current reproduction as an adaptive response to a perceived decreased likelihood in survival and future reproduction. Males increased sperm transfer to females by as much as 3.7-fold, which strongly supports the idea of a major shift in the trade-off between current and future reproductive investment resulting in an ‘all outʼ adaptive current reproductive effort strategy. The more stressed the males were, the more sperm they transferred, in an attempt to maximize their lifetime reproductive success. Similar adaptive responses have been observed in other insects in response to stressors similarly associated with uncertainty in male future reproductive reproduction. In *Drosophila*, increased perception of male-male competition is a biotic stressor known to affect a maleʼs investment in current reproduction in the form of increased mating duration and sperm transfer [41, 42]. In the moth *Ephestia kuehniella*, the same stimulus resulted in larger ejaculates and this increase in current investment resulted in decreased future reproductive success and lifespan [43].

In the mosquito *An. coluzzii*, hydric stress has been shown to have important consequences on metabolic reserves [21]. Thus, it may be that changes in metabolic reserves are the cues for the maleʼs adaptive reproductive investment response rather than ambient humidity itself. Another unknown is the exact relationship between increased sperm cells transfer and current reproductive success. Male ejaculate amount correlates with female fecundity in a wide variety of arthropod taxa including the Diptera [44], but the exact relationship between the amount of sperm transferred to females and female fecundity in *An. coluzzii* remains to be investigated. Consequently, we currently do not know the exact extent of the benefits in terms of current reproductive success that stressed males may gain from their larger investment. Male *An. coluzzii* can only inseminate ~ 3 females per night and are limited in the amount of sperm and the size of the mating plug they can transfer per female [24]. Therefore, for the stressed males that increased their sperm ejaculate so drastically, it probably means that they mated once only, thus giving an opportunity for a larger number of males to contribute to the insemination of females. This would underline not only a change in male sperm transfer strategy but also in their mating behaviour, with possible impact on female mate choice and, generally, the sexual selection processes.

In contrast to males, females exposed to hydric stress prior to mating did not significantly increase the amount of sperm taken up into the spermatheca. However, the rate of insemination was 13% lower than in the reciprocal male experiment. Since females depend on future blood meals to produce egg-batches, they are expected to promote their body maintenance to maximize their chance of surviving until successfully feeding on a host. Mating bears a small but significant fitness cost on females [28] and may be associated with significant predation risks in nature. This could explain the fact that female *An. coluzzii* evolved to be largely monandrous. In this experiment this mating cost may also explain why fewer females were receptive to mating and delayed mating altogether.

In *An. coluzzii*, female monandry is the rule which also suggest that the amount of sperm taken up by females *via* a single mating event must usually be adequate for the femaleʼs future reproductive needs [23]. In previous studies, we found very variable quantities of sperm cells stored in the spermathecae [24]. Here, this was again the case, with numbers ranging from ~ 1700 to 7000. Taken together with the even wider stress-induced variation observed in the stressed-male experiment, these results do not support the idea that females can actively regulate the initial amount of sperm they take up in their spermathecae following mating. Hence, whilst females may decide not to mate, once they have been inseminated the regulation of sperm cell numbers for long term storage and maintenance must occur whilst the sperm is already lodged within the spermatheca.

When sperm cells reach the female spermatheca *via* the spermathecal duct, they are maintained and nourished *via* specialized cells of the epithelium lining the inside of the female spermatheca [45]. In *Drosophila melanogaster,* studies focusing on sperm selection process have shown a decrease in sperm viability within a couple of weeks after mating [46]. In *An. coluzzii*, sperm viability needs to be maintained for much longer durations because females sometimes need to store sperm for several months of estivation during the dry season before reinitiating oogenesis when the rainy season begins [47]. The extent to which hydric stress affects how females regulate sperm numbers through sperm maintenance in the initial days following mating was the focus of the 7-day post-mating stress experiment. Interestingly, we found a much lower number of sperm cells 7 days post-mating across all treatment groups compared to the two pre-mating experiments. Given the standard rearing protocol and environmental conditions used across all three experiments, this suggest that females may actively get rid of excess sperm. A fast decrease in the proportion of viable sperm measured in the female reproductive tract has also been observed in the dung fly, *Scathophaga stercoraria*, and *D. melanogaster* [46, 48]. Further experiments in that species indicate that some sperm are maintained for longer amounts of times and that this amount depends on food predictability [49]. Here, the activity of the remaining sperm in our experimental groups did not significantly differ between hydric stress levels, albeit there was a trend for sperm activity to decrease in the high stress group and female survival was significantly impacted in that group. The results suggest that over the first 7 days post-mating, females do not significantly trade sperm maintenance against overall body maintenance and survival [47, 50] but that those effects may occur over a longer stress period.

## Conclusions

Overall, the results of this study lend support to the life-history prediction which stipulates that in a situation of life-threatening environmental uncertainty, males should increase their current reproductive effort. Here, this translated into a stark increase in sperm transferred to females. Facing similar conditions, females are predicted to prioritize survival till an opportunity for blood feeding and laying eggs arises. Here we observed a decrease in mating rates which may be compatible with a prioritization of body maintenance and survival, whilst females that mated maintained a low but viable sperm number independent of stress level. Future work should attempt to measure the fitness costs and benefits from these stress-induced strategies directly through detailed measurements of realized reproductive success such as egg batch size and emergence rates and adult lifespan until death.

## Data Availability

All data generated or analysed during this study are included in this published article.

## Abbreviations

ANOVA: analysis of variance
CT: cycle threshold
PBS: phosphate-buffered saline
PCR: polymerase chain reaction
qPCR: quantitative polymerase chain reaction
